# Variation of sentinel lymphatic channels (SLCs) and sentinel lymph nodes (SLNs) assessed by contrast-enhanced ultrasound (CEUS) in breast cancer patients

**DOI:** 10.1186/s12957-017-1195-3

**Published:** 2017-07-10

**Authors:** Ying Wang, Wenbin Zhou, Cuiying Li, Haiyan Gong, Chunlian Li, Nianzhao Yang, Xiaoming Zha, Lin Chen, Tiansong Xia, Xiaoan Liu, Minghai Wang, Qiang Ding

**Affiliations:** 1 Department of General Surgery, The First Affiliated Hospital with Nanjing Medical University, 300 Guangzhou Road, 210029 Nanjing, China; 2 Department of Ultrasound, The First Affiliated Hospital with Nanjing Medical University, 300 Guangzhou Road, 210029 Nanjing, China; 3 Department of General Surgery, The First Affiliated Yijishan Hospital with Wannan Medical College, Wuhu, Anhui China

**Keywords:** Breast cancer, Contrast-enhanced ultrasound, Sentinel lymphatic channel, Sentinel lymph node

## Abstract

**Background:**

The objective of this study was to assess the feasibility of detecting the variation of sentinel lymphatic channels (SLCs) and sentinel lymph nodes (SLNs) in breast cancer patients using contrast-enhanced ultrasound (CEUS).

**Methods:**

A total of 46 breast cancer patients were prospectively recruited in the study. All the participants received intradermal and peritumoral injection of microbubbles as contrast agent, and SLCs and SLNs were assessed preoperatively. Blue dye was injected subareolarly and peritumorally during the surgery. The SLNs detected by CEUS and blue dye were sent to the pathology laboratory for histopathological analysis.

**Results:**

At least one SLC and SLN were detected by CEUS in all 46 cases. Three types of SLCs were detected, including superficial sentinel lymphatic channels (SSLCs), penetrating sentinel lymphatic channels (PSLCs), and deep sentinel lymphatic channels (DSLCs). Five lymphatic drainage patterns (LDPs) were found, including SSLC, PSLC, SSLC + PSLC, SSLC + DSLC, and SSLC + PSLC + DSLC. Only SSLC was detected on CEUS in 24 cases; only PSLC was detected in 3 cases; both SSLC and PSLC were detected in 8 cases; both SSLC and DSLC were detected in 7 cases; SSLC, PSLC, and DSLC were all detected in the remaining 4 cases. An actual LDP was defined on the combination of CEUS and dissection of the specimen. The accuracy rate of CEUS was 43/46. Interestingly, a bifurcated SLC was found in 8 patients. In 3 patients, a discontinuous SLC and non-enhanced SLN were found by CEUS. Also, no dyed SLNs were detected during the surgery. The axillary lymph nodes turned out tumor involved histologically.

**Conclusion:**

CEUS is feasible to assess the variation of SLCs and SLNs preoperatively in breast cancer patients. SLNB is not suggested when a discontinuous SLC and non-enhanced SLN were detected by CEUS.

## Background

Sentinel lymph node (SLN) is the first node in the lymphatic system to receive lymphatic drainage [1]. Sentinel lymph node biopsy (SLNB) has become a standard surgical technique in the management of early invasive breast cancer patients with clinically negative lymph node as it can reduce postoperative morbidity compared with axillary lymph node dissection [2].

However, the lymphatic drainage of the breast has not been studied clearly yet. Sappey has investigated lymphatic drainage of breast in 1874, observed that the lymph of the breast collected in a subareolar plexus and then drained towards the axilla through lymph collection vessels [3, 4]. Sappey’s description of breast lymphatic drainage has been universally accepted for nearly 100 years. However, in 1959 Turner-Warwick has found that the breast drained directly from the tumor to the axilla [3, 5]. In our previous study [6], three types of sentinel lymphatic channels (SLCs) have been found, including superficial sentinel lymphatic channels (SSLCs), penetrating sentinel lymphatic channels (PSLCs), and deep sentinel lymphatic channels (DSLCs). SSLCs originate from the subareolar lymphatic plexus and pass within the subcutaneous fatty tissue; PSLCs originate from the subareolar lymphatic plexus and penetrate through the breast parenchyma; DSLCs originate from parenchyma and pass through the breast parenchyma or within the retromammary cellular space. Therefore, based on these three types of SLCs, varied lymphatic drainage patterns (LDPs) could be formed draining the breast to the axilla, which could make an influence on the accuracy of SLNB [6].

Based on previous studies [3–5] and our own findings about SLCs [6], the number of SLCs and SLNs are varied among breast cancer patients, which can partly explain why the number of SLNs is an important factor which would affect the accuracy of SLNB [7–9]. Surgeons with little experience may miss some SLNs during the surgery [10]. So, evaluating the SLCs and SLNs preoperatively could be of important significance. A large amount of studies have been conducted aiming at assessing SLNs preoperatively. The methods included computed tomography, magnetic resonance imaging, single-photon emission computed tomography, and fluorescence [11–14]. Since Mattrey RF [15] and his colleagues firstly applied contrast-enhanced ultrasound (CEUS) on SLN detection in breast cancer patients, it has been constantly studied because of its ease of use and cost effectiveness [16–23]. In the study conducted by Sever AR and colleagues, SLCs and SLNs could be detected preoperatively with a high sensitivity [23], allowing doctors to observe the enhancement of SLCs and SLNs in real time. They have found the existence of SLCs using CEUS, but the location and number of SLCs have not been reported, while the variation of SLCs among breast cancer patients would make an influence on the accuracy of SLNB [3–6].

In this study, we aimed to assess the feasibility of evaluating the variation of SLCs and SLNs preoperatively using CEUS in order to improve the accuracy of SLNB.

## Methods

### Patient enrollment

Between November 2015 and December 2016, 46 consecutive patients were recruited into the study prospectively. Inclusion criteria included (1) invasive breast cancer diagnosed by core biopsy, (2) no axillary lymph node involvement by physical examination, and (3) patients went to undergo modified radical mastectomy. Exclusion criteria included (1) multiple tumor, (2) preoperative chemotherapy, (3) previous axillary node dissection, and (4) severe medical comorbidities

### CEUS examination

CEUS was performed to the enrolled patients by two experienced sonographers (Gong H, Li C) using a MyLab™ Twice scanner (Esaote, Genoa, Italy). A high frequency linear-array probe (LA522) was used. Low mechanical index values were applied (0.05) to reduce the destruction of a contrast agent. A gray-scale ultrasound examination of the axilla was carried out before the injection of the contrast agent. The microbubbles (SonoVue™ BRACCO Imaging, S.p.A, Milan, Italy) used as a contrast agent was reconstituted with 3 ml of saline (NaCl 0.9%). Using a 25-G needle for local anesthesia, 3 ml of 2% lidocaine was injected into the subcutaneous layer of the areola. Using a tuberculin syringe with a 25-GA 5/8 needle, 1 ml of the reconstituted microbubbles was injected intradermally into the skin immediately adjacent to the mammary papillae [21]. The infiltration of microbubbles into the SSLC/PSLC and the superficial sentinel lymph nodes (SSLNs)/penetrating sentinel lymph nodes (PSLNs) could be observed dynamically on CEUS. After the contrast agent drained out, 2 ml of microbubbles was injected into a single area of the peritumoral parenchyma approaching the axilla. The infiltration of microbubbles into the DSLC and the deep sentinel lymph nodes (DSLNs) could also be observed dynamically on CEUS. Immediately after the examination, all the enhanced SLNs were marked with a titanium clip (BARD, Ultra CLIP, San Geronimo, Humacao, USA) under the ultrasound guidance. After the titanium clip was inserted, gray-scale ultrasound examination was conducted to confirm the position of the clip, which was shown with high-echo.

### Dissection of the excised specimen after surgery and pathological analysis

Under general anesthesia, 2 ml of blue dye was injected in the same site as microbubbles have been injected. The injection site was massaged for 5 min before the modified radical mastectomy. After the surgery, the excised specimen was examined carefully and all the dyed SLCs were dissected. Then, all the axillary lymph nodes (ALNs) underwent an X-ray examination to identify the titanium clip-marked SLNs which were enhanced on CEUS. If the titanium clip-marked SLNs found by X-ray examination were also dyed, it meant that the CEUS examination and blue dye came with the same result. All blue dyed SLNs, titanium clip-marked SLNs, and other ALNs were sent to the pathology laboratory for further histopathological analysis. If any suspicious cells were noted, immunohistochemical staining for cytokeratin was applied.

### Statistical analysis

Mean, ratio, and range were analyzed for continuous variables. An actual LDP was defined on the combination of both CEUS and blue dye findings. The accuracy of CEUS on LDP assessment was determined as the ratio of the number of patients in which the LDP defined by CEUS coincided with actual LDP to the number of total participants.

## Results

### Baseline characteristics

In all, 46 patients were enrolled. CEUS was conducted before modified radical mastectomy. The clinical characteristics of these 46 patients are shown in Table 1. The mean age was 50.5 years, with a range of 35–66 years. The mean tumor size was 2.5 cm, with a range of 1.5–4.5 cm. Thirty-one tumors were located in the lateral-superior quadrant of the breast, 6 located in the interior-superior quadrant, 7 located in the lateral-inferior quadrant, and the remaining 2 located in the central part. Thirty-one were ER/PR positive, 16 were HER2 overexpressed, and 6 were triple negative.

**Table 1:** Characteristics of patients and breast tumors

Variables	Number (%)
Age	
Median (range)	50.5 (35–66)
Tumor size	
≤2 cm	16
2–5 cm	30
Tumor location	
Lateral-superior	31
Interior-superior	6
Lateral-inferior	7
Central	2
Receptor status	
ER/PR positive and HER2 negative	24
ER/PR negative and HER2 positive	9
ER/PR positive and HER2 positive	7
Triple negative	6

### LDPs found by CEUS examination

**Fig. 1:**
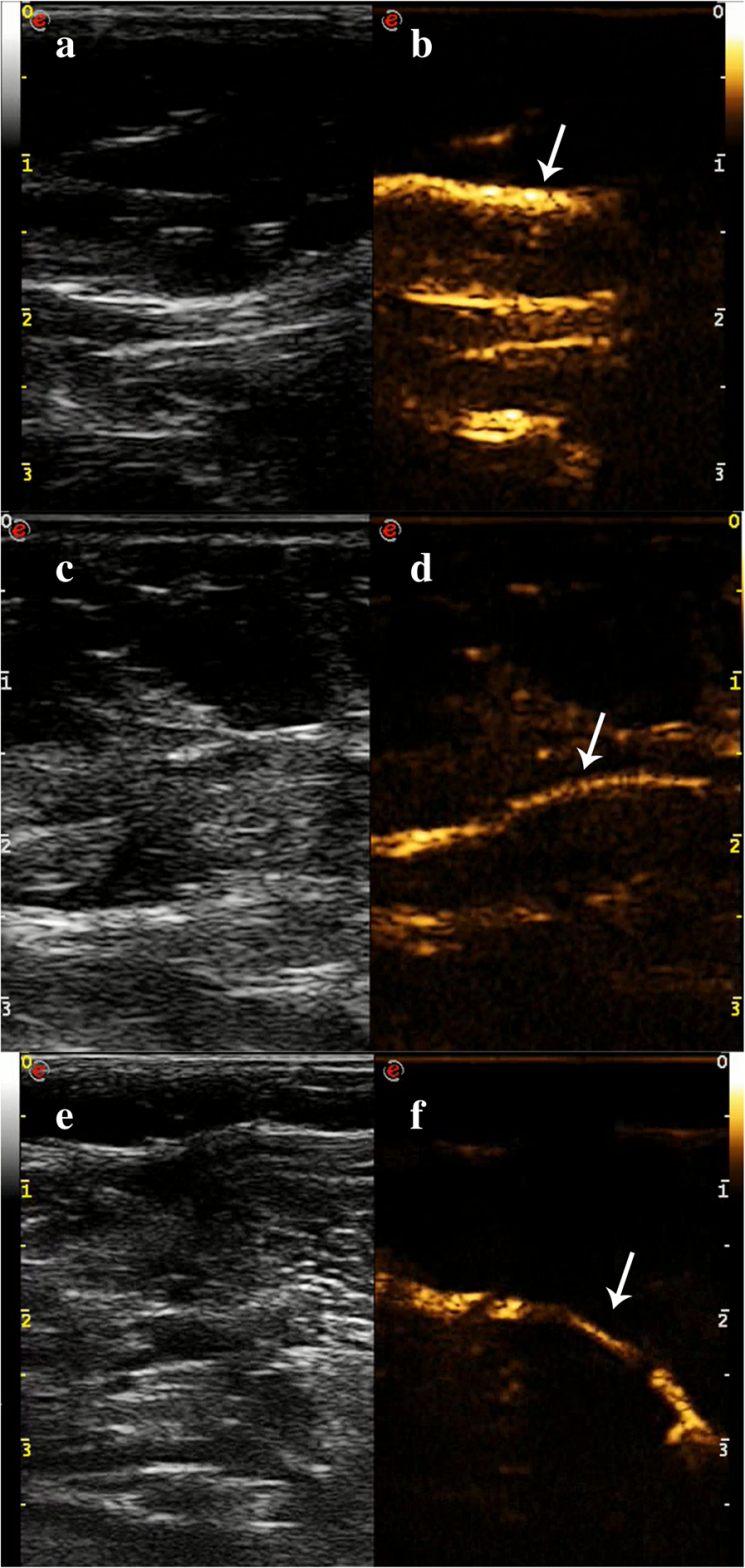
Three kinds of SLCs shown on live dual images. Live dual images can provide images of gray-scale ultrasound and CEUS simultaneously, which could help to identify the enhanced SLCs. Three kinds of SLCs were observed on live dual images in real time: a SSLC which could not be observed on a gray-scale imaging (**a**) but was enhanced on a contrast-specific imaging (**b**, *arrow*); a PSLC which could not be observed on a gray-scale imaging (**c**) but was enhanced on a contrast-specific imaging (**d**, *arrow*); a DSLC which could not be observed on a gray-scale imaging (**e**) but was enhanced on a contrast-specific imaging (**f**, *arrow*)

**Fig. 2:**
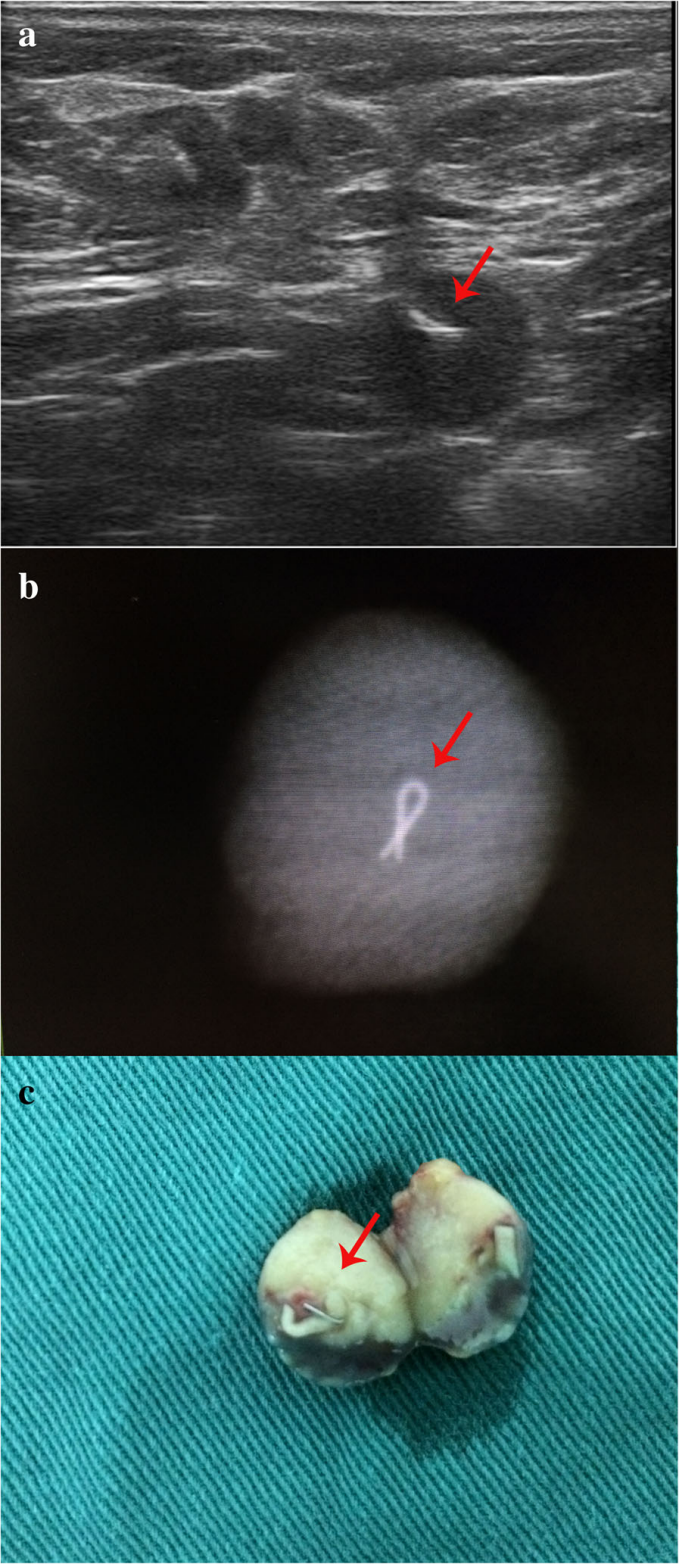
Gray-scale ultrasound examination, X-ray examination, and dissection used to confirm the insertion of a titanium clip into the SLN. **a** The titanium clip (*arrow*) was shown with high-echo on gray-scale ultrasound. **b** The titanium clip (*arrow*) was shown with high density on X-ray imaging. **c** The titanium clip (*arrow*) can be viewed macroscopically after dissecting the dyed SLN

**Fig. 3:**
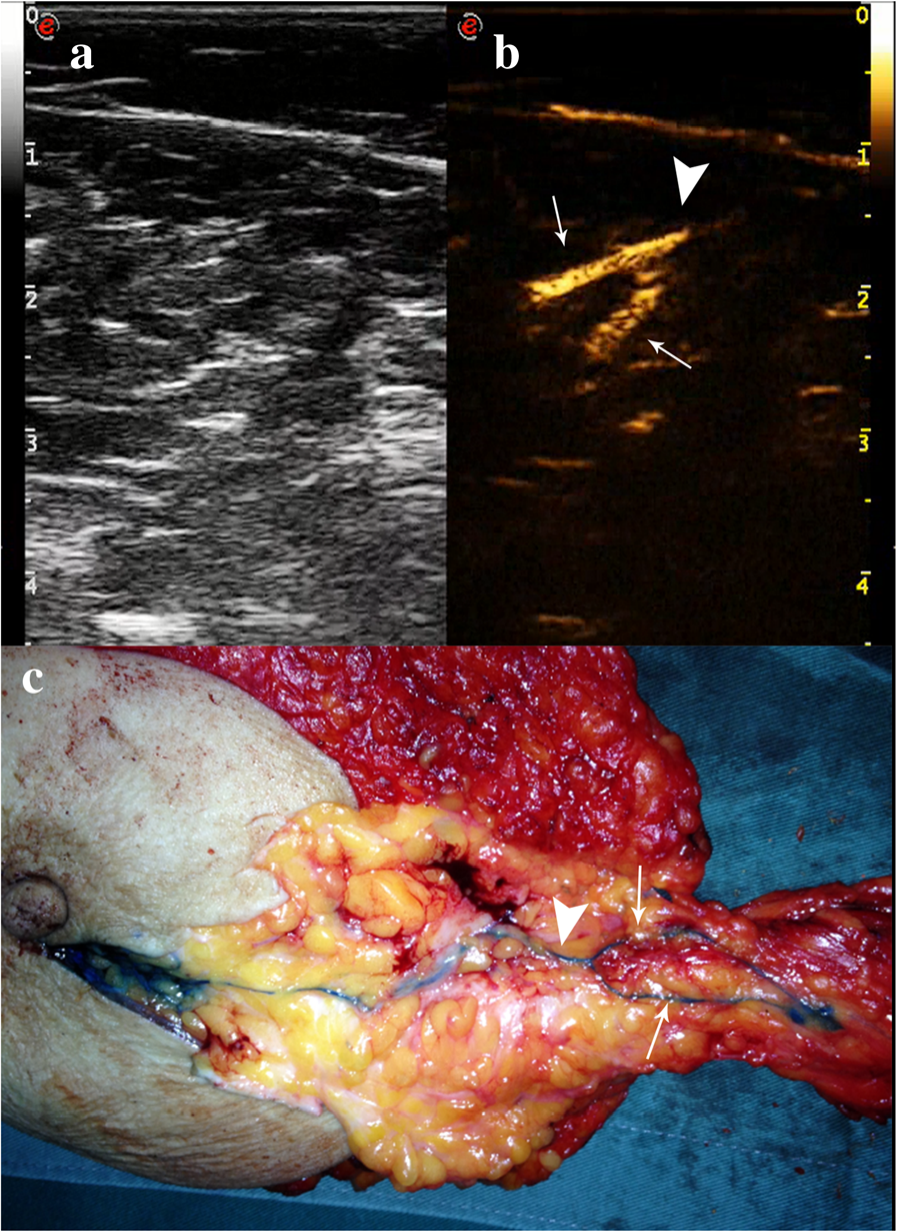
Live dual images and macroscopic appearance of a bifurcated SLC. **a** The bifurcated SLC could not be observed on gray-scale imaging. **b** The bifurcated SLC with one trunk (*arrowhead*) and two branches (*arrow*) were enhanced on CEUS. **c** The bifurcated SLC with one trunk (*arrowhead*) and two branches (*arrow*) could be macroscopically observed

**Fig. 4:**
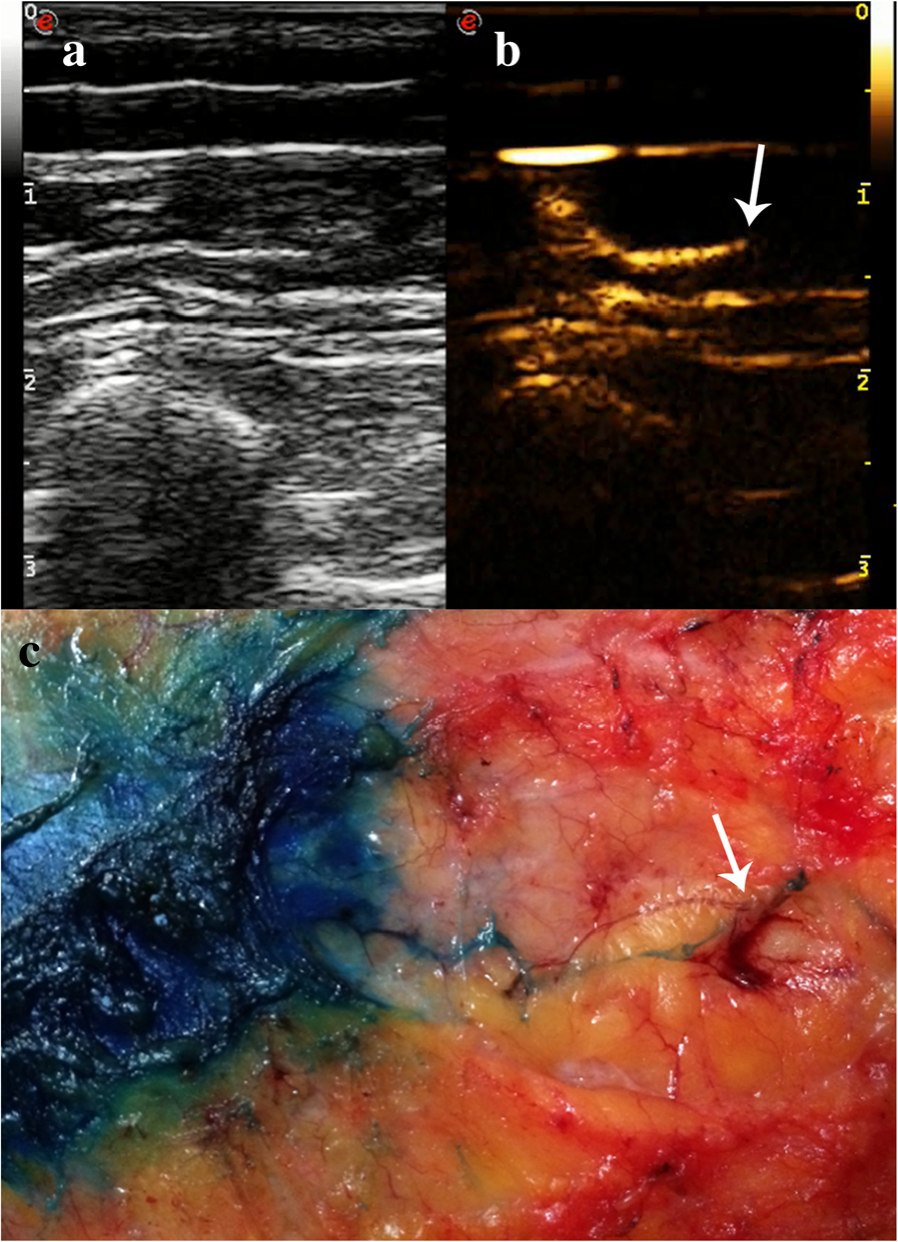
Live dual images and macroscopic appearance of a discontinuous SLC. **a** The discontinuous SLC could not be observed on gray-scale imaging. **b** The discontinuous SLC was shown to get interrupted (*arrow*) on CEUS. **c** The SLC was discontinuously dyed (*arrow*)

In all 46 cases, at least one SLC was detected on CEUS. In this study, three types of SLCs, including SSLC, PSLC, and DSLC, were detected by CEUS (Fig. 1). All the enhanced SLNs were marked with titanium clips successfully (Fig. 2). Five LDPs, including SSLC, PSLC, SSLC + PSLC, SSLC + DSLC, and SSLC + PSLC + DSLC, were found: only SSLC was detected in 24 cases; only PSLC in 3 cases; both SSLC and DSLC in 7 cases; both SSLC and PSLC in 8 cases; SSLC, PSLC, and DSLC in the remaining 4 cases (Table 2). Interestingly, SLCs were found bifurcated in 8 patients (Fig. 3). A discontinuous SLC without corresponding SLN enhancement was found in 3 cases (Fig. 4).

**Table 2:** The LDPs defined by CEUS and actual LDPs^a^

LDP	CEUS	Actual LDP	Blue dye
SSLC	24	21	21
PSLC	3	3	3
SSLC + PSLC	8	8	8
SSLC + DSLC	7	10	10
SSLC + PSLC + DSLC	4	4	4

^a^Actual LDPs: defined basing on both CUES and blue dye results

### Actual LDPs defined on the combination of both CEUS and blue dye findings

An actual LDP was defined based on the findings of CEUS and dissection of specimen, which is shown on Table 2: only SSLC was detected in 21 cases; only PSLC was detected in 3 cases; both SSLC and DSLC were detected in 10 cases, both SSLC and PSLC were detected in 8 cases; SSLC, PSLC, and DSLC were all detected in the remaining 4 cases.

There were 43 cases in which the LDPs found by CEUS were consistent with actual LDPs. For all the 3 inconsistent cases, DSLCs were not enhanced on CEUS.

### Histopathological results of SLNs detected by CEUS and other ALNs

The number of SLNs detected by CEUS ranged from 1 to 4. The histopathological results of SLNs detected by CEUS and other ALNs are presented on Table 3. There were 3 inconsistent cases, in which the DSLCs and DSLNs were not enhanced on CEUS, the enhanced SSLNs, non-enhanced DSLNs, and other ALNs were all non-metastatic. In the 43 consistent cases, the SLNs detected by CEUS were found metastatic in 11 cases. There were 4 false negative cases, in which the SLNs detected by CEUS were non-metastatic while metastatic lymph nodes were found among other ALNs. In 3 of the false negative cases, a discontinuous SLC and non-enhanced SLN were found.

**Table 3:** Histopathological results of the SLNs detected by CEUS and other ALNs

	Number	SLNs	Other ALNs
Inconsistent cases	3	−	−
Consistent cases	28	−	−
	8	+	+
	3	+	−
	4	−	+

## Discussion

In this study, we detected three types of SLCs and five LDPs using CEUS. The detection rate of SLC was 46/46, and the accuracy was 43/46. For all the 3 inconsistent cases, DSLCs were missed. It may be due to the size of microbubbles and the less lymph capillaries in the parenchyma [24]. The contrast microbubbles are encapsulated gas bubbles smaller than red blood cells with a diameter range 0.7–10 μm [16], which is larger than the endothelial gap. So, sufficient massage is needed after the injection of microbubbles. Moreover, a new contrast agent with a smaller size may improve the detection accuracy of CEUS.

Robert F [15] and his colleagues first applied microbubbles on SLN detection in breast cancer patients. They found that the infiltration of lymphatic channels could be observed in real time after the subcutaneous injection of a contrast agent. Another study conducted by Sever AR [23] reported that the sensitivity of CEUS on SLN detection was 96%. They noticed the existence of SLCs, but the location and number of SLCs were not reported. In this study, three types of SLCs and five LDPs were detected by CEUS successfully, including SSLC, PSLC, SSLC + PSLC, SSLC + DSLC, and SSLC + PSLC + DSLC. The detection rate was 46/46. The accuracy was 43/46. In previous studies, the number of SLNs was 1 or 2 when the contrast agent was injected in the areola area only [15–23]. In this study, 4 patients were found with 3–4 SLNs. This may result from the variation of SLCs as more than one SLC existed and some were bifurcated. This result suggests that CEUS may be a feasible way to assess the number of SLNs preoperatively.

The SLC was found bifurcated in 8 patients. Two branches drained to two different SLNs. In some cases, the SLN of each branch was located closely; otherwise, they could be spatially far apart, one of the SLNs may be located superficially while the other one is located quite deep in the axilla. In this situation, it may be with a high possibility to miss one of the two SLNs during the SLNB. So, using CEUS to assess the SLC and SLN preoperatively may be of important significance.

A discontinuous SLC was found in three cases, metastatic ALNs were found in all these cases. In two of these cases, the SLCs were also discontinuously dyed, and the corresponding SLNs were not dyed, either. This may partly explain the false negative result of SLNB using blue dye. Same result was also obtained by Goldberg when administrating microbubbles in a melanoma tumor animal model [25]. We presumed that the SLC is embolized by tumor cells. So, SLNB is not suggested in this situation as it may lead to a false negative result.

Our study has some limitations. First, studies with more participants should be conducted to assess the impact of CEUS examination on a false negative rate of SLNB. Second, the titanium clips used for marking the enhanced SLNs were too small to be viewed or touched intraoperatively, which may cause difficulties in clinical application, whereas a guidewire may be of practical value.

## Conclusion

CEUS is feasible to assess the variation of SLCs and SLNs preoperatively in early breast cancer patients. A discontinuous SLC and non-enhanced SLN on CEUS may be a sign of SLN metastasis, SLNB is not suggested in this situation. Clinical studies with more participants are still needed to confirm our findings. A new contrast agent with a smaller size and better enhanced effect could be developed to improve the detection accuracy of CEUS.

## Abbreviations

ALN: Axillary lymph node
CEUS: Contrast-enhanced ultrasound
DSLC: Deep sentinel lymphatic channel
DSLN: Deep sentinel lymph nodes
LDP: Lymphatic drainage patterns
PSLC: Penetrating sentinel lymphatic channel
PSLN: Penetrating sentinel lymph node
SLC: Sentinel lymphatic channel
SLN: Sentinel lymph node
SLNB: Sentinel lymph node biopsy
SSLC: Superficial sentinel lymphatic channel
SSLN: Superficial sentinel lymph node.
